# Glyphosate induces benign monoclonal gammopathy and promotes multiple myeloma progression in mice

**DOI:** 10.1186/s13045-019-0767-9

**Published:** 2019-07-05

**Authors:** Lei Wang, Qipan Deng, Hui Hu, Ming Liu, Zhaojian Gong, Shanshan Zhang, Zijun Y. Xu-Monette, Zhongxin Lu, Ken H. Young, Xiaodong Ma, Yong Li

**Affiliations:** 1 Department of Cancer Biology, Lerner Research Institute, Cleveland Clinic, Cleveland, OH USA; 2 School of Life Sciences, Institute of Modern Aquaculture Science and Engineering, Guangdong Provincial Key Laboratory for Healthy and Safe Aquaculture, South China Normal University, Guangzhou, 510631 China; 3 Department of Medical Laboratory, Central Hospital of Wuhan, Wuhan, China; 4 State Key Laboratory of Respiratory Diseases, Guangzhou Institute of Respiratory Diseases, The First Affiliated Hospital of Guangzhou Medical University, Guangzhou Medical University, Guangzhou, China; 5 Department of Stomatology, the Second Xiangya Hospital, Central South University, Changsha, China; 6 Department of Stomatology, Xiangya Hospital, Central South University, Changsha, China; 7 Department of Hematopathology, The University of Texas MD Anderson Cancer Center, Houston, TX USA; 8 Institute for Brain Research and Rehabilitation, South China Normal University, Guangzhou, 510631 China; 9 The Research Center of Basic Integrative Medicine, Guangzhou University of Chinese Medicine, Guangzhou, 510006 China

**Keywords:** Glyphosate, Multiple myeloma, Vk*MYC mice, Activation-induced cytidine deaminase

## Abstract

**Background:**

Glyphosate is the most widely used herbicide in the USA and worldwide. There has been considerable debate about its carcinogenicity. Epidemiological studies suggest that multiple myeloma (MM) and non-Hodgkin lymphoma (NHL) have a positive and statistically significant association with glyphosate exposure. As a B cell genome mutator, activation-induced cytidine deaminase (AID) is a key pathogenic player in both MM and B cell NHL.

**Methods:**

Vk*MYC is a mouse line with sporadic MYC activation in germinal center B cells and considered as the best available MM animal model. We treated Vk*MYC mice and wild-type mice with drinking water containing 1000 mg/L of glyphosate and examined animals after 72 weeks.

**Results:**

Vk*MYC mice under glyphosate exposure developed progressive hematological abnormalities and plasma cell neoplasms such as splenomegaly, anemia, and high serum IgG. Moreover, glyphosate caused multiple organ dysfunction, including lytic bone lesions and renal damage in Vk*MYC mice. Glyphosate-treated wild-type mice developed benign monoclonal gammopathy with increased serum IgG, anemia, and plasma cell presence in the spleen and bone marrow. Finally, glyphosate upregulated AID in the spleen and bone marrow of both wild-type and Vk*MYC mice.

**Conclusions:**

These data support glyphosate as an environmental risk factor for MM and potentially NHL and implicate a mechanism underlying the B cell-specificity of glyphosate-induced carcinogenesis observed epidemiologically.

**Electronic supplementary material:**

The online version of this article (10.1186/s13045-019-0767-9) contains supplementary material, which is available to authorized users.

## Introduction

Glyphosate is the most popular and profitable agrochemical, being registered to use in over 160 countries and accounting for around 25% of the global herbicide market. It acts via inhibition of 5-enolpyruvylshikimate-3-phosphate synthase (EPSPS) in the shikimate pathway, which is critical to the growth of most plants but absent in animals. Since the discovery of this herbicidal activity in 1974, glyphosate usage has increased enormously, particularly with the recent introduction of genetically modified crops carrying a glyphosate-resistant version of EPSPS. Glyphosate is also heavily used in crop pre-harvest desiccation. Glyphosate has been detected in more than 50% of surface waters in the USA, with a median concentration of ~ 0.02 μg/L and a maximum concentration of 427 μg/L [1]. Around agricultural basins, the median levels of glyphosate range from 0.08 to 4.7 μg/L, with the highest detected concentration of 430 μg/L [2]. Beyond surface water, glyphosate is found in soil, air, and groundwater, as well as in food [3]. In a recent report, urinary excretion levels of glyphosate among older residents of Rancho Bernardo, CA, where glyphosate use is significantly lower than in the US Midwest region, increased from 0.024 to 0.314 μg/L from 1993 to 2016 [4].

Multiple epidemiological studies have investigated the association of glyphosate exposure and cancer risk using either cohort or case-control designs [5]. These studies found no significant association between glyphosate exposure and overall cancer risk but suggested that glyphosate exposure is positively associated with multiple myeloma (MM) and non-Hodgkin lymphoma (NHL), as concluded by a working group of the International Agency for Research on Cancer (IARC), the cancer agency of the World Health Organization (WHO) [5]. In contrast, other national and international agencies like the US Environmental Protection Agency (EPA), European Food Safety Authority, European Chemicals Authority, and the Joint Food and Agriculture Organization of the United Nations and WHO have maintained that glyphosate is unlikely to pose a carcinogenic risk [6]. Three case-control studies performed in Iowa [7], France [8], and Canada [9] suggest that glyphosate exposure increases MM risk. The most recent update (2018) from the Agricultural Health Study, however, found no association between glyphosate exposure and either MM or NHL [10]. Such inconsistencies likely reflect unidentified confounders, recall bias, and the complex nature of human exposure that impact epidemiologic relationships, underscoring the importance of investigations using animal models to test the effects of exposures in a controlled environment. However, neither mouse nor rat studies have been reported that specifically examine the impact of glyphosate in the pathogenesis of MM, which is one of the two cancer types relevant to humans reported to be associated with glyphosate exposure thus far.

A hallmark of MM is that virtually all MM cases are preceded by monoclonal gammopathy of undetermined significance (MGUS) [11]*.* Bergsagel and colleagues generated a mouse model of MM (Vk*MYC) under the C57bl/6 genetic background with sporadic c-Myc activation in germinal center B cells, resulting in the development of benign monoclonal gammopathy, a mouse equivalent to MGUS, which then progresses to MM. This is the best available MM animal model because it recapitulates many biological and clinical features of human MM, including increased serum immunoglobulin G (IgG), bone lesions, and kidney damage [12]. In this work, we used Vk*MYC mice to test our hypothesis that glyphosate has a pathogenic role in MM.

## Materials and methods

### Mouse model and treatments

**Fig. 1:**
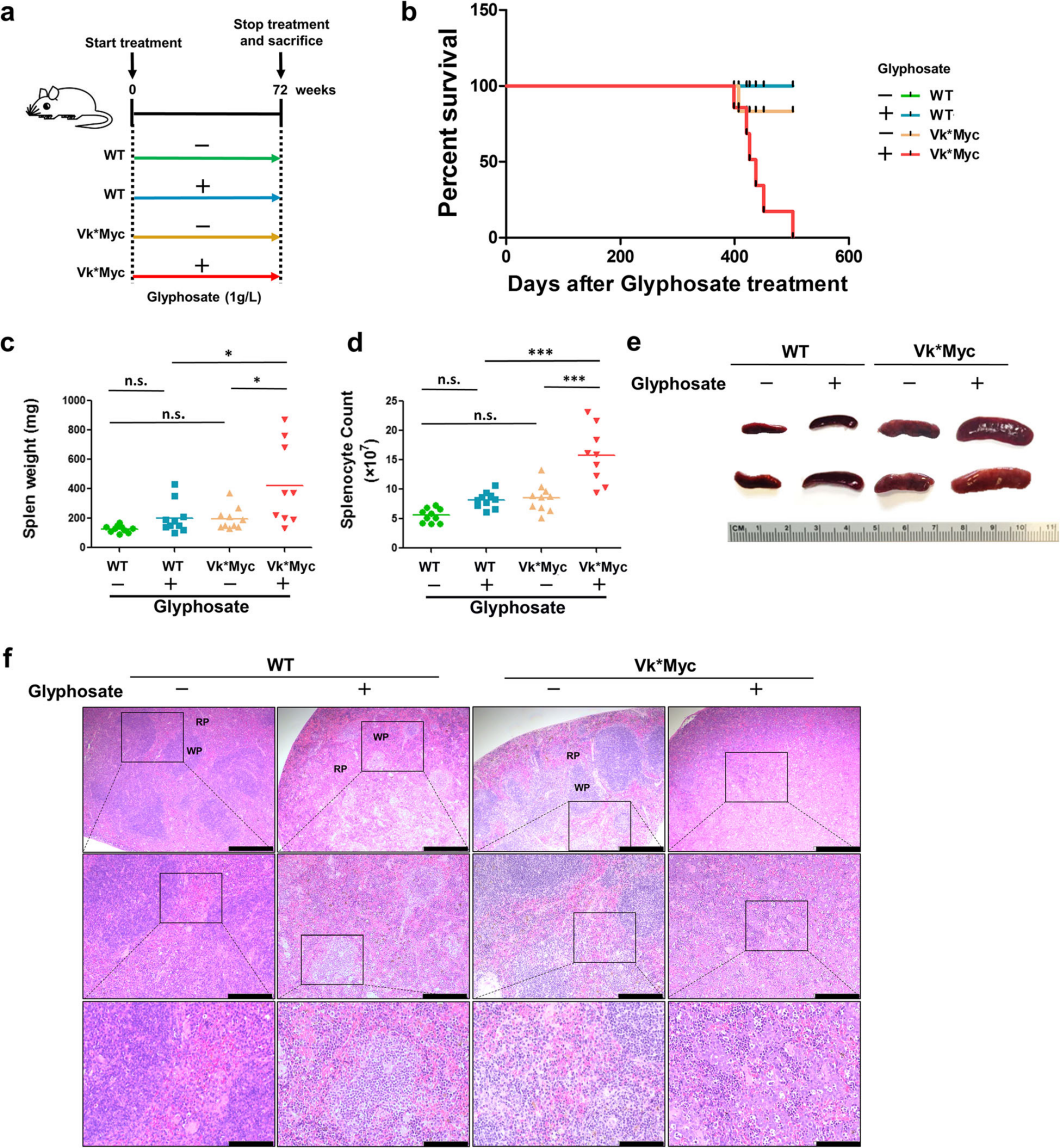
Glyphosate reduced survival and induced splenomegaly in Vk*MYC mice. **a** Schematic diagram of the chronic glyphosate exposure regimen in 4 groups of mice. **b** The percentage of mice surviving under glyphosate exposure. The line (blue) to indicate untreated WT mice aligned directly with that for WT treated mice and so was not visible. **c** Mouse spleen weight at sacrifice. **d** The total number of splenocytes per spleen from mice at sacrifice. **e** Representative images of spleens from 4 groups (2 per group). **f** The spleens from control and glyphosate-treated mice were fixed, embedded in paraffin, sectioned, stained with H&E, and examined by light microscopy. Representative H&E-stained spleen sections from glyphosate-treated Vk*MYC mice showing altered architecture with a reduction of lymphoid white pulp (WP) and an expansion of hematogenous red pulp (RP). Scale bar = 500 μm (top), 200 μm (middle), or 100 μm (bottom). Data in **c** and **d** were analyzed by one-way ANOVA (spleen from one treated Vk*MYC animal was not included due to an incidental damage). The horizontal lines indicate the mean value. *n* = 10 mice per group. *P ≤ 0.05; **P ≤ 0.01; ***P ≤ 0.001

All chronic and acute animal experiments were performed in accordance with NIH guidelines and under protocols approved by the Cleveland Clinic Institutional Animal Care and Use Committee. Wild-type (WT) C57Bl/6 mice were purchased from the Jackson Laboratory (Bar Harbor, ME). Vk*MYC mice in the C57Bl/6 genetic background were obtained from Dr. Leif Bergsagel (Mayo Clinic, Scottsdale, AZ) [12]. Vk*MYC and WT mice were intercrossed to obtain WT and Vk*MYC littermates. Sex-matched WT and Vk*MYC mice (8 weeks old) were assigned to treatment or control groups based on body weight. For chronic study of glyphosate effects, treatment groups were provided 1.0 g/L glyphosate (Sigma-Aldrich, St. Louis, MO) in their drinking water for 72 weeks. Regular drinking water was provided for the control groups (Fig. 1a). Every 6 weeks, blood was collected from the tail vein of mice, and the serum IgG level was measured. We did not monitor the serum concentration of glyphosate for mice due to sample availability. However, the gross amount of drinking water consumed by each group of studied mice was monitored and no difference was observed between these groups. Animal regulations prevented us from maintaining mice till they died of cancer (natural death). Instead, mice had to be euthanized whenever they reached humane endpoints (i.e., adverse health deterioration and serious complications). Treated Vk*MYC mice began to reach humane endpoints starting at week 60 with 4 surviving until week 66 and 3 surviving to week 71. At week 72, the remaining 3 surviving Vk*MYC mice reached humane endpoints. These 3 treated Vk*MYC mice were used for M-spike detection and pathologic analyses, along with mice from other groups. Other Vk*MYC mice that were sacrificed before week 72 were analyzed for total serum IgG levels, complete blood cell count, and total serum creatinine. For comparison, mice from other groups were euthanized at week 72 and their tissues and blood analyzed. For acute treatment, 8-week-old mice (*n* = 5 per group) were given 0, 1.0, 5.0, 10.0, or 30.0 g/L of glyphosate for 7 days before sacrifice. The same variables were analyzed in the acute study.

### Blood and post-mortem assays

Whole-blood complete blood count (CBC), IgG enzyme-linked immunosorbent assay (ELISA), serum protein electrophoresis, flow cytometry, and histological examinations of relevant tissues were performed as described previously [13]. Serum creatinine was measured by ELISA using a creatinine assay kit (#ab65340, Abcam, Cambridge, MA) according to the manufacturer’s protocol.

### Western blotting analyses

Mouse tissues were processed for Western blotting as we have described elsewhere [13]. The antibodies were from Cell Signaling Technology (Danvers, MA, USA): AID (L7E7) (#4975) and β-actin (#3700). Blotting was run with 3 technical replicates. Horseradish peroxidase-conjugated anti-rabbit or anti-mouse IgG was used as the secondary antibody.

### Statistics

Statistical analysis was carried out using GraphPad InStat 3 software (GraphPad Software, Inc., San Diego, CA, USA). The statistical significance between the groups was determined by one-way or two-way analysis of variance (ANOVA) with the appropriate post hoc testing using Tukey’s test. Statistical significance was accepted at *P* ≤ 0.05. All data are shown as mean ± SEM unless otherwise indicated.

## Results

### Chronic glyphosate exposure reduces survival and induces splenomegaly in Vk*MYC mice

Eight-week-old Vk*MYC mice and their WT littermates were provided 1.0 g/L glyphosate in drinking water for 72 weeks, and animals were monitored at regular intervals before sacrifice (Fig. 1a). Glyphosate significantly affected the health of Vk*MYC mice, all of which had to be euthanized by week 72 (Fig. 1b). Surviving mice in other groups were sacrificed at week 72 (at age 80 weeks) for necropsy. Inspection of organs revealed a marked increase in spleen weight and size in Vk*MYC mice treated with glyphosate compared to the other 3 groups (Fig. 1c, e). Glyphosate significantly augmented the splenocyte number in Vk*MYC mice (Fig. 1d). Histopathologic analysis revealed distinct red and white pulp in the spleens of untreated WT and Vk*MYC mice, suggesting normal splenic organization. These histological characteristics were not preserved in the spleens from WT mice treated with glyphosate, with predominant red pulp involvement and poorly organized white pulp. The spleens from Vk*MYC mice challenged with glyphosate showed hematogenous red pulp without lymphoid white pulp involvement, with more vacuoles and lymphocyte necrosis. Additionally, marked histological disorganization such as severe splenorrhagia was observed in some areas, which blurred the boundaries between red pulp and white pulp (Fig. 1f). These findings indicate that glyphosate induces splenomegaly in both WT and Vk*MYC mice.

### Hematological abnormalities occur in Vk*MYC mice with chronic glyphosate exposure

**Fig. 2.**
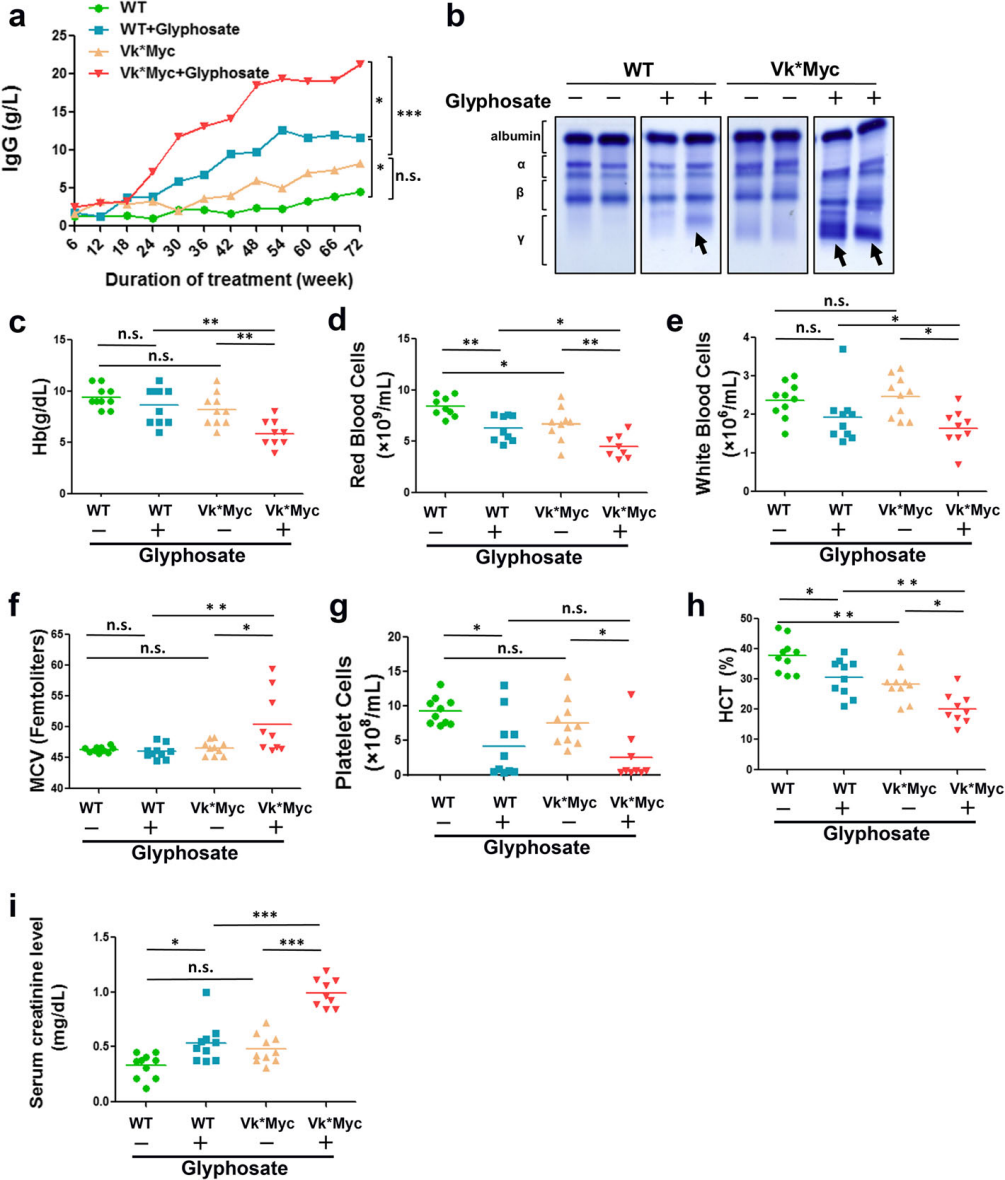
Hematological abnormalities found in Vk*MYC mice treated with glyphosate. **a** Total serum IgG in mice during 72 weeks of glyphosate treatment. Mouse blood samples were collected and assayed for IgG every 6 weeks. **b** Immunoglobins from mice as determined by SPEP at week 72. Arrows indicate IgG clonal peaks (M-spike; γ-globulin peak). SPEP was performed for all mice in each group, and representative results of 2 mice per group are shown. **c**–**h** Complete blood cell counts in mice. Hemoglobin concentration (Hb, **c**), red blood cell count (**d**), white blood cell count (**e**), mean red cell volume (MCV, **f**), platelet cell count (**g**), and hematocrit (HCT, **h**) are shown. **i** Total serum creatinine in mice at week 72. The horizontal lines indicated the mean value. Data were analyzed by two-way ANOVA (**b**) or one-way ANOVA (**a**, **d**, **e**). *n* = 10 mice per group

As illustrated in Fig. 2a, untreated Vk*MYC mice exhibited higher IgG levels than untreated WT mice. Upon glyphosate exposure, WT mice showed moderate yet steady increasing in IgG levels, suggesting that glyphosate induces benign monoclonal gammopathy, a mouse equivalent to human MGUS. Vk*MYC mice receiving glyphosate had greater IgG elevation, and by week 30, IgG levels jumped to 11.78 g/L, more than 5-fold the 2.07 g/L observed in untreated Vk*MYC mice. From week 36 to week 72, the mean IgG level was significantly higher in treated WT and Vk*MYC mice compared to the untreated control groups, and Vk*MYC mice, treated or untreated, had higher IgG levels than their WT counterparts (Additional file 1: Figure S1). Overt MM diagnosis was determined by serum protein electrophoresis (SPEP) analysis to detect the M-spike, which is a significant IgG monoclonal peak. SPEP results showed that Vk*MYC mice treated with glyphosate had a clear M-spike, whereas weaker M-spike was observed in glyphosate-treated WT mice. No clear M-spike was present in the untreated WT mice or Vk*MYC mice (Fig. 2b). This is the direct in vivo evidence that glyphosate exposure leads to M-spike, a cardinal hematological abnormality consistent with MM.

Hematological abnormalities were present in glyphosate-treated mice as compared to untreated control mice (Fig. 2c–i). The hemoglobin concentration was significantly lower in glyphosate-treated Vk*MYC mice than in untreated Vk*MYC mice or glyphosate-treated WT mice. Glyphosate treatment slightly decreased the red blood and white blood cell counts and increased mean red cell volume in Vk*MYC mice compared with WT mice. The platelet counts and hematocrit were also reduced in glyphosate-treated Vk*MYC mice. Serum creatinine level is a marker for kidney function, with higher levels indicating kidney dysfunction. In glyphosate-treated Vk*MYC mice, the mean serum creatinine concentration was 0.99 mg/dL, about 2-fold of that in untreated Vk*MYC mice (0.48 mg/dL) and treated WT mice (0.53 mg/dL). These data support the notion that glyphosate induces multiple hematological abnormalities characteristic of MM in mice.

### Vk*MYC mice chronically exposed to glyphosate develop progressive plasma cell neoplasms

**Fig. 3:**
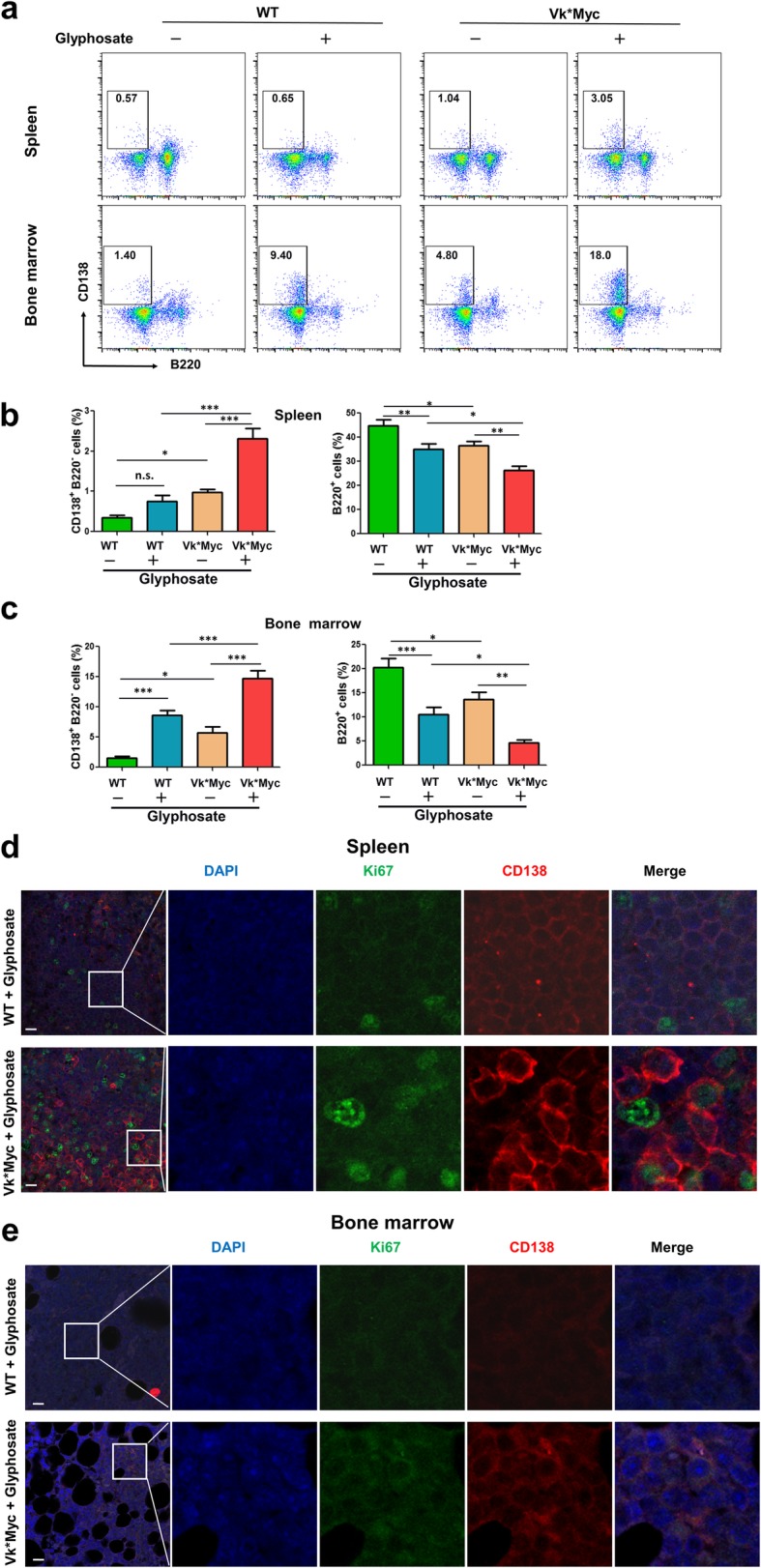
Glyphosate-treated Vk*MYC mice developed progressive plasma cell neoplasms. **a** Representative flow cytometry plots detecting cell surface markers CD138 (*Y*-axis) and B220 (*X*-axis) in splenocytes (upper panel) and bone marrow cells (lower panel). The numbers on the axes denoted the log_10_ values of fluorescence. The numbers in the inserts show the percentage of CD138^high^B220^-^ cells in the entire cell population. **b**, **c** Bar graphs of the percentages of CD138^+^B220^-^ and B220^+^ cells from the spleen (**b**) and bone marrow (**c**). Data were analyzed by one-way ANOVA. **d** Confocal microscopy images identifying Ki67^+^ (green) and CD138^+^ (red) expression with nuclear DAPI staining of cells in the spleen of a representative WT (upper panel) and Vk*MYC mouse (lower panel), both treated with glyphosate. Scale bar = 10 μm. **e** Confocal microscopy images identifying Ki67^+^ (green) and CD138^+^ (red) expression with nuclear DAPI staining of cells in the bone marrow of WT (upper panel) and Vk*MYC mice (lower panel) treated with glyphosate. Scale bar = 10 μm. *n* = 10 mice per group. *P ≤ 0.05; **P ≤ 0.01; ***P ≤ 0.001

Plasma cells exhibit CD138^hi^ B220^–^ (high CD138 expression without B220 expression). Flow cytometric analyses of cells harvested from the spleens and bone marrow showed expansion of plasma cells in mice under glyphosate exposure. A marked increase in the numbers of CD138^hi^ B220^–^ cells was detected in both WT and Vk*MYC mice treated with glyphosate (Fig. 3a). Glyphosate-treated Vk*MYC mice averaged 2.3% CD138^hi^ B220^–^ plasma cells in the spleen, which was significantly higher than the 0.98% in untreated Vk*MYC mice and the 0.76% in treated WT mice (Fig. 3b). Remarkably, the bone marrow of glyphosate-treated WT and Vk*MYC mice harbored approximately 8.6% and 14.7% CD138^hi^ B220^–^ plasma cells, respectively, significantly higher than their untreated counterparts (Fig. 3c).

To assess plasma cell localization and compartmentalization in the spleen and bone marrow, we stained tissue sections using antibodies against CD138^+^ (plasma cells) and Ki67^+^ (a marker for proliferation). The number of plasma cells was greater in both spleen and bone marrow of treated Vk*MYC mice compared to treated WT mice (Fig. 3d, e). In the spleens of Vk*MYC mice, most plasma cells stained weakly for Ki67, indicating that these cells were not plasmacytoma cells, which are generally proliferative. These data demonstrate that glyphosate treatment expands the plasma cell population in the spleen and bone marrow in both WT and Vk*MYC mice.

### Chronic glyphosate exposure triggers multiple organ dysfunction

**Fig. 4:**
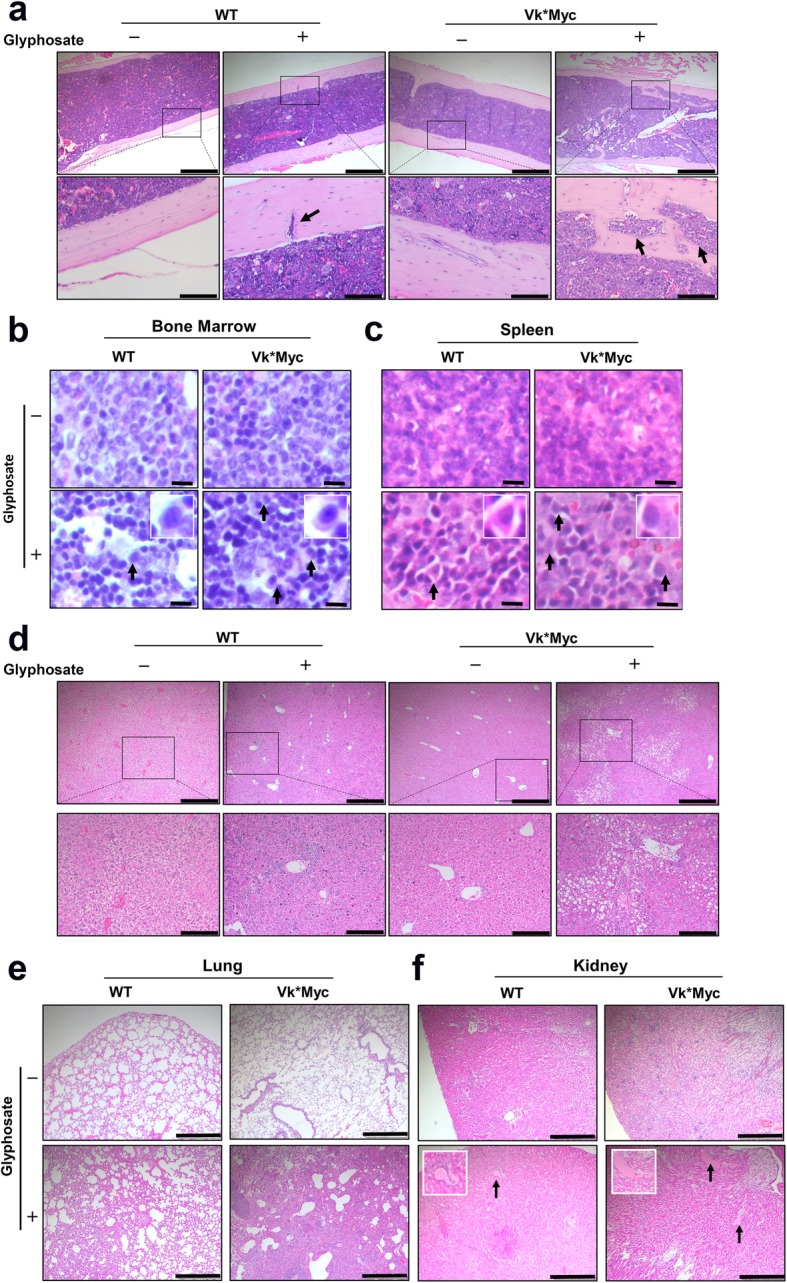
Glyphosate led to multiple organ dysfunction. **a** Histological evaluation of bone morphology from 4 groups of mice. Bone lytic lesions (indicated by arrows) were detected in the femoral shaft of Vk*MYC mice treated with glyphosate. Scale bar = 500 μm (top) or 100 μm (bottom). **b** Infiltrating plasma cells in the bone marrow of glyphosate-treated mice. Scale bar = 20 μm. Arrows pointed to plasma cells. **c** Infiltrating plasma cells in the spleen of glyphosate-treated mice. Scale bar = 20 μm. Arrows point to plasma cells. **d** Collagen deposition in the liver was observed in glyphosate-treated Vk*MYC mice. *n* = 10 mice per group. Scale bar = 500 μm (top) or 200 μm (bottom). **e** Destruction of lung morphology was observed in glyphosate-treated Vk*MYC mice. *n* = 10 mice per group. Scale bar = 500 μm. **f** Protein deposition (indicated by arrows) in the kidney was observed in glyphosate-treated Vk*MYC mice. *n* = 10 mice per group. Scale bar = 500 μm. All panels show 1 representative image each from 4 groups of mice unless otherwise indicated

To determine whether target organ damage occurred in glyphosate-treated mice, the femoral shaft, spleen, liver, lung, and kidney were sectioned and stained with hematoxylin and eosin (H&E). Severe destructive osteolytic bone lesions in the femoral shaft were readily detectable in glyphosate-treated Vk*MYC mice. Treated WT mice showed smaller bone lesions. No lesions were observed in the control groups (Fig. 4a). Plasma cells with a perinuclear clear zone and eccentric round nucleus were observed in glyphosate-treated WT and Vk*MYC mice (Fig. 4b, c).

Next, we analyzed the histopathologic changes in the liver, lung, and kidney. In glyphosate-treated mice, hepatic fibrosis and collagen deposition were observed in Vk*MYC mice, whereas WT mice showed less severe liver damage; the 2 control groups had normal hepatic tissue architectures (Fig. 4d). The lungs in treated Vk*MYC mice were severely damaged, with large distal air spaces filled by lymphocytes, neutrophils, cell debris, and hyperplastic pneumocytes; those from untreated WT mice had normal alveolar spaces and alveolar septa lined with normal pneumocytes. The lungs from treated WT mice and untreated Vk*MYC mice showed an intermediate phenotype (Fig. 4e). Renal tubular obstruction by large casts, indicative of necrotic tubular cells, were detected in glyphosate-treated WT and Vk*MYC mice, but not in the untreated groups; there were more and larger casts in treated Vk*MYC kidneys than in WT kidneys (Fig. 4f). Taken together, these data indicate that glyphosate treatment damages multiple organs in both WT and Vk*MYC mice with more severe damage occurring in Vk*MYC mice.

### Chronic glyphosate exposure induces AID upregulation

**Fig. 5:**
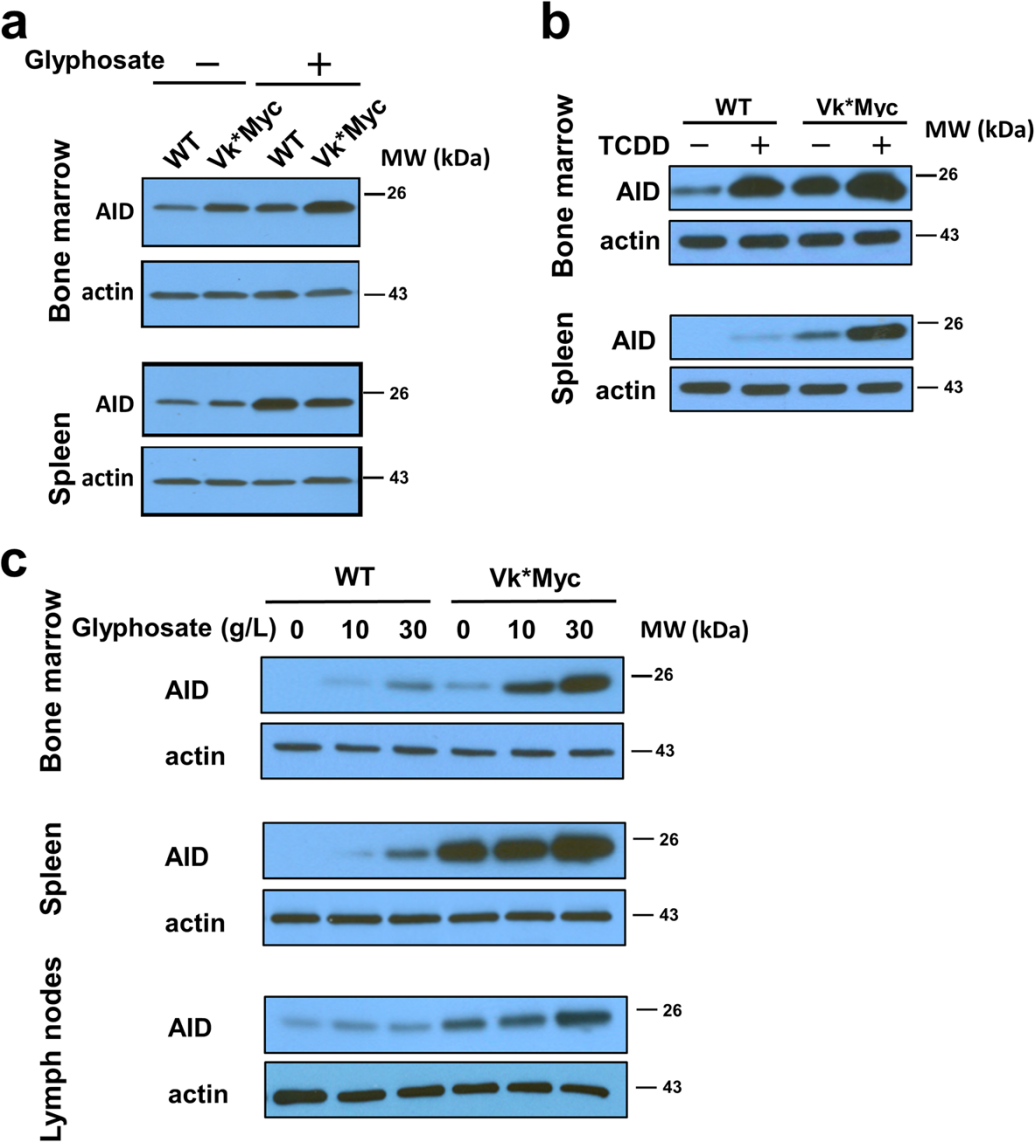
Glyphosate-induced AID upregulation. **a** Western blotting analysis of mice treated with 1.0 g/L of glyphosate for 72 weeks. **b** Western blotting analysis of mice treated with TCDD. **c** Western blotting analysis of mice treated with glyphosate for 7 days. One representative mouse per treatment group is shown

To investigate the underlying mechanisms of glyphosate-mediated MGUS induction and MM progression, we determined the expression of activation-induced cytidine deaminase (AICDA, also known as AID) in mice treated with 1.0 g/L glyphosate for 72 weeks. We found that AID was upregulated in both the bone marrow and the spleen of WT and Vk*MYC mice (Fig. 5a). For untreated animals, AID expression was moderately higher in the bone marrow of Vk*MYC mice. In our previous study [13], we found that 2,3,7,8-tetrachlorodibenzo-p-dioxin (TCDD), a contaminant in Agent Orange, induced MGUS in WT mice and promoted MM progression in Vk*MYC mice. We examined the expression of AID in WT and Vk*MYC mice treated with TCDD chronically [13]. TCDD increased AID expression in both bone marrow and spleen of both WT and Vk*MYC mice (Fig. 5b).

### Acute glyphosate exposure induces AID upregulation

To determine the acute effect of glyphosate, we treated 8-week-old WT and Vk*MYC mice with increasing doses of glyphosate (1, 5, 10, and 30 g/L) in drinking water for 7 days. This acute treatment neither increased spleen weight nor affected body weight significantly. Only at the highest dose (30 g/L, Additional file 1: Figure S2a–c) did WT and Vk*MYC mice have a detectable M-spike and significantly higher serum IgG (Additional file 1: Figure S2d). The serum creatinine level was not significantly affected (Additional file 1: Figure S2e). The plasma cell populations in the bone marrow, spleen, and lymph node of WT and Vk*MYC mice were moderately increased in the treated groups (Additional file 1: Figure S3). Next, we analyzed the expression of AID in the spleen, bone marrow, and lymph nodes and found that AID was upregulated in a glyphosate dose-dependent manner in the spleen and bone marrow of WT and Vk*MYC mice treated with 10 and 30 g/L of glyphosate (Fig. 5c). AID was highly expressed in the spleen of untreated Vk*MYC mice but was highest with 30 g/L glyphosate treatment. AID expression in lymph nodes was only higher in Vk*MYC mice treated with 30 g/L glyphosate. Lower doses (1 and 5 g/L) did not upregulate AID expression in any organs of WT or Vk*MYC mice (data not shown). For untreated animals, AID expression in the spleen, bone marrow, and lymph nodes was higher in Vk*MYC mice than that in WT mice, in agreement with previous results showing that MYC transcriptionally upregulates AID expression [14]. It is notable that the basal AID level in these acute treatment groups differed from that in the chronic glyphosate study, likely due to the difference in ages at measurement (9 weeks versus 80 weeks).

Given the role of AID in MM pathogenesis in the context of its capacity to induce mutations and chromosome translocations [12, 15, 16], these results from mice with chronic and acute glyphosate treatment support an AID-mediated mutational mechanism in the etiology of MGUS and MM under glyphosate exposure.

## Discussion

We have reviewed 9 studies testing glyphosate as a single agent for carcinogenicity in either mice (2 studies) or rats (7 studies) via chronic dietary or drinking water administration (Additional file 2: Table S1). Both mouse studies showed a positive trend toward increased incidence of some rare cancers (kidney tumor [17–19] or hemangiosarcoma [20]) in male, but not female, CD-1 mice exposed to the highest doses of glyphosate. Of the 7 rat studies, 4 (including 1 in which animals received drinking water ad lib containing 2700 mg/L glyphosate for 24 months [21]) found no significant increase in cancer incidence in any groups of treated animals [20]. Two other rat studies reported increased pancreas adenoma incidence in males treated with intermediate glyphosate doses; however, animals receiving the highest doses developed these tumors at a lower incidence than those receiving the intermediate doses [22–25] (Additional file 2: Table S1). The last rat study is quite controversial, scientifically and otherwise. Seralini et al. (2012) reported that female Sprague-Dawley rats receiving 400 mg/L glyphosate in drinking water for 24 months had an increased mammary tumor incidence (100%) compared to the no-glyphosate control (50%), yet the incidence was 90% for the 2250 mg/L group [26]. Many challenged the pathological and statistical analysis of this study [27, 28]. The study was retracted [29], but some alleged the retraction was influenced by the agrochemical giant Monsanto (acquired by Bayer AG) [30], a major manufacturer of both glyphosate and glyphosate-resistant genetically modified crop seeds. The authors (2014) then republished this study without further review [31]. Largely based on the results from these rodent studies and multiple epidemiological studies, the IARC concluded that “there is sufficient evidence in experimental animals for the carcinogenicity of glyphosate” [5], whereas the EPA, European Food Safety Authority, European Chemicals Agency, and the Joint Food and Agriculture Organization of United Nations and WHO Meeting on Pesticide Residues (JMPR) concluded otherwise [6]. Specifically, JMPR stated that “administration of glyphosate […] at doses as high as 2000 mg/kg body weight by the oral route, the route most relevant to human dietary exposure, was not associated with genotoxic effects in an overwhelming majority of studies conducted in mammals” [20].

Our literature review, however, identifies a major drawback in these studies—these strains of mice and rats generally do not develop MM, which is one of the only two cancers that are linked to glyphosate exposure in epidemiological studies. The availability of the Vk*MYC mouse model, widely regarded as the best animal model for MM, has allowed us to make the first direct determination of whether glyphosate contributes to MM pathogenesis [12]. In this study, we demonstrate that glyphosate induces benign monoclonal gammopathy (mouse equivalent to MGUS in human) in WT mice and promotes MM progression in Vk*MYC mice*.* In Vk*MYC mice, glyphosate causes hematological abnormalities like anemia and multiple organ dysfunction like lytic bone lesions and renal damage, which are hallmarks of human MM. We examined the lymph nodes located in armpits, groin, and neck of treated mice and found no tangible lymphomas by week 72. Yet, we cannot exclude the possibility that glyphosate may accelerate lymphomagenesis in WT mice if longer glyphosate exposure is applied.

Beyond epidemiology and animal models, the mechanism of action is the third pillar required to define a compound as a carcinogen. Numerous studies have revealed that glyphosate may induce DNA damage, oxidative stress, inflammation, and immunosuppression, as well as modulate cell proliferation and death and disrupt sex hormone pathways [5]. However, these mechanistic studies have failed to explain why glyphosate exposure is only positively associated with MM and NHL. Our results demonstrate that glyphosate treatment, either at a chronic low dose or acute high doses, upregulates the expression of AID in the bone marrow and spleen of both WT and Vk*MYC mice. AID is a B cell-specific genome mutator [15] and a key pathogenic player in both MM [12] and B cell lymphoma [16], with the latter accounting for ~ 90% of NHL cases. Specific to MM, the early genetic events are dominated by translocations involving the *IgH* locus, which are probably generated via abnormal somatic hypermutation and class switch recombination mediated by AID. We also noted that TCDD, a contaminant the herbicide Agent Orange, also upregulates AID expression (Fig. 5). Our data disclose, for the first time, that glyphosate elicits a B cell-specific mutational mechanism of action in promoting carcinogenesis, as well as offering experimental evidence to support the epidemiologic finding regarding its tissue specificity in carcinogenesis (i.e., only increasing the risk for MM and NHL).

The “acceptable daily intake (ADI)” of glyphosate currently allowed in the USA, defined as the chronic reference dose as determined by EPA, is 1.75 mg/kg body weight/day [32]; an average adult male or female in the USA who weighs 88.8 or 76.4 kg [33] and drinks 2 L (8 glasses) water daily containing 77.7 (for male) or 66.9 (for female) mg/L glyphosate would reach the ADI. In a previous study, rats subjected to 2700 mg/L glyphosate for 24 months did not have a significantly higher cancer incidence (Additional file 2: Table S1). Therefore, we chose a dose of *1*,*000 mg*/*L* glyphosate in drinking water (~ 15-fold the ADI) in this study, which caused significant adverse effects and accelerated MM progression in Vk*MYC mice, i.e., animals predisposed to MM. We are cognizant that an individual would unlikely consume such an excessive dose of glyphosate; however, our results are of regulatory importance and suggest that the ADI for glyphosate should be reassessed, particularly for certain populations, such as MGUS patients.

## Conclusions

Our data provide in vivo evidence to support that glyphosate induces MGUS and promotes disease progression to MM. We uncover a B cell-specific mutational mechanism for glyphosate exposure that increases MM and NHL risk, providing a molecular basis for human epidemiological findings. Given the increasing use of glyphosate in the USA and worldwide, the present study supports epidemiological reports and informs the EPA and other agencies during the regulatory development of current and emerging glyphosate-based herbicidal products.

## Additional files

Additional file 1: **Figures S1** and **S2.** Supplementary figures. (PDF 1473 kb)

Additional file 2: **Table S1.** Supplementary table. (PDF 37 kb)

## Data Availability

All data and materials supporting the conclusion of this study have been included within the article and the supplemental data.

## Abbreviations

ADI: Acceptable daily intake
AID: Activation-induced cytidine deaminase
ANOVA: Analysis of variance
CBC: Complete blood count
ELISA: Enzyme-linked immunosorbent assay
EPA: Environmental Protection Agency
EPSPS: 5-Enolpyruvylshikimate-3-phosphate synthase
H&E: Hematoxylin and eosin
IgG: Immunoglobin G
JMPR: The Joint Food and Agriculture Organization of United Nations and WHO Meeting on Pesticide Residues
MGUS: Monoclonal gammopathy of undetermined significance
MM: multiple myeloma
NHL: Non-Hodgkin lymphoma
SPEP: Serum protein electrophoresis
TCDD: 2,3,7,8-Tetrachlorodibenzo-p-dioxin
WHO: World Health Organization
WT: Wild-type
