# Supplementary material for: Glyphosate induces benign monoclonal gammopathy and promotes multiple myeloma progression in mice
Source: J Hematol Oncol. 2019 Jul 5;12:70. doi: 10.1186/s13045-019-0767-9 (PMC6612199; doi:10.1186/s13045-019-0767-9)
Supplement: Supplementary file 1 — Figures S1 and S2. Supplementary figures. (PDF 1473 kb) [file 13045_2019_767_MOESM1_ESM.pdf]

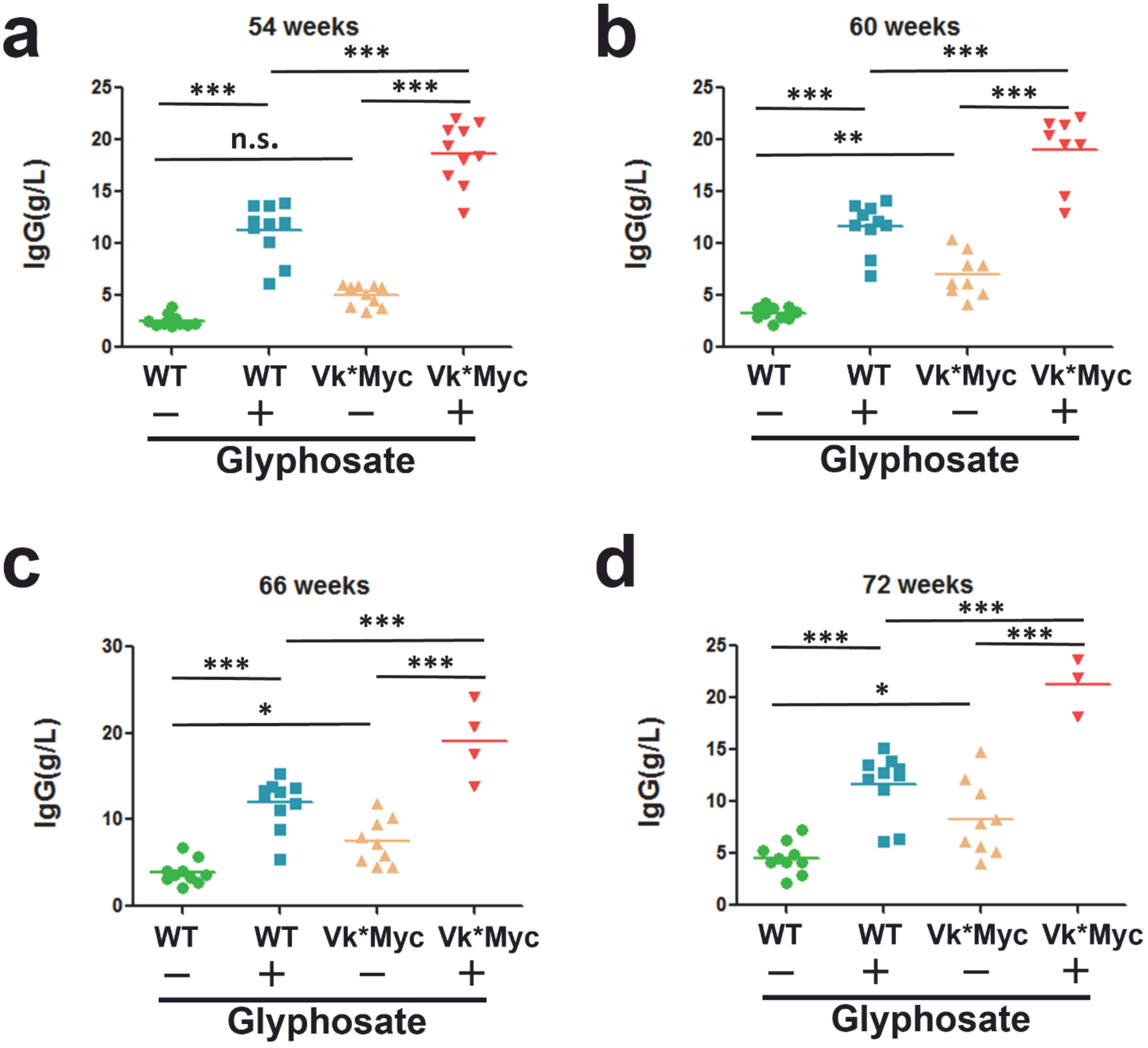

Figure S1. Total serum IgG levels in mice. Serum IgG levels were determined by ELISA at Week 54 (a), 60 (b), 66 (c), and 72 (d). n = 10 mice per group. There were only 4 and 3 live animals in the treated Vk*Myc group by Week 66 and 72, respectively, so these weeks have IgG readings only from these mice. *, $P \leq 0.05$; **, $P \leq 0.01$; ***, $P \leq 0.001$.

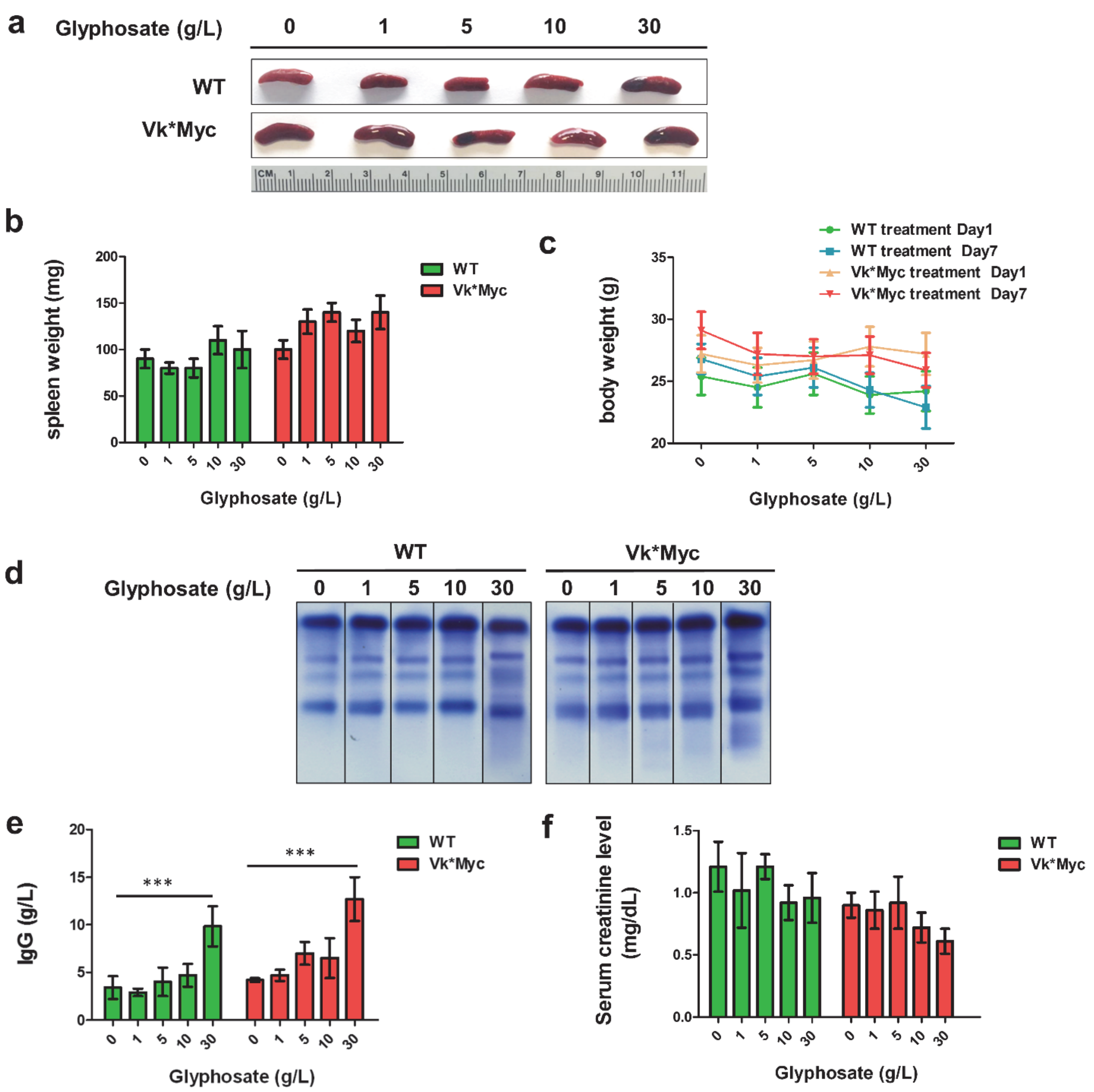

Figure S2. Splenic and hematological phenotypes of animals with acute glyphosate exposure. (a) Images of representative spleens from each group. (b) Spleen weight. (c) Body weight. (d) M-spike. (e) Serum IgG levels. (f) Serum creatinine levels. ***, $P < 0.001$. $n = 5$ mice per group

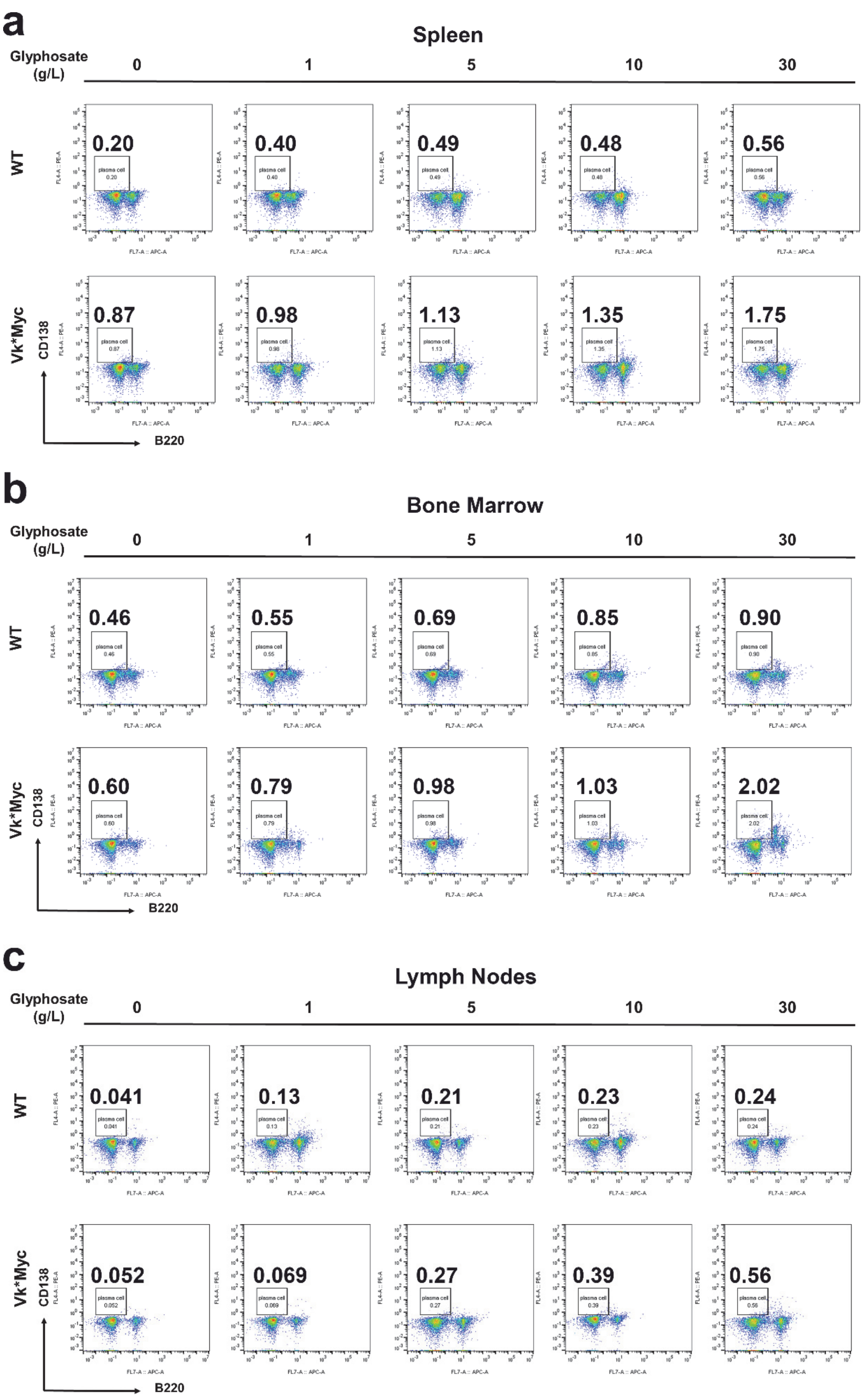

Figure S3. Flow cytometry analysis of plasma cells in mice acutely treated with glyphosate. Cells from spleen (a), bone marrow (b), and lymph nodes (c) of mice were analyzed for plasma cells ($CD138^{+}B220^{-}$). The percentages of plasma cells were shown on the top of the inserts. n = 5 mice per group.
