# Supplementary material for: Glyphosate induces benign monoclonal gammopathy and promotes multiple myeloma progression in mice
Source: J Hematol Oncol. 2019 Jul 5;12:70. doi: 10.1186/s13045-019-0767-9 (PMC6612199; doi:10.1186/s13045-019-0767-9)
Supplement: Supplementary file 2 — Table S1. Supplementary table. (PDF 37 kb) [file 13045_2019_767_MOESM2_ESM.pdf]

Supplemental Table 1 Summary of rodent studies of glyphosate and cancer incidence

Animal (No. per group) *	Treatment **	Duration	Results ***
**CD-1 mice (n =50 M and 50 F)**	Diet, 0, 1,000, 5,000, or 30,000 ppm	24 months	Renal tubule adenoma or carcinoma (6% for the 30,000 ppm M group$^{\Delta}$)
**CD-1 mice (n =50 M and 50 F)**	Diet, 0, 100, 300, or 1,000 ppm	24 months	Hemangiosarcoma (8% for the 1,000 ppm M group$^{\Delta}$)
**S-D rats (n =10 M and 10 F)**	Water, 0, 5.0 x 10^{-5}, 400, or 2,250 mg/L	24 months	Mammary tumors (100% in the 400 mg/L F group$^{\Delta}$, 50, 90, and 90% for 0, 5.0 x 10^{-5}, and 2,250 mg/L F groups)
**S-D rats (n =60 M and 60 F)**	Diet, 0, 2,000, 8,000, or 20,000 ppm	24 months	Pancreas islet cell adenoma (14% in the 2,000 ppm M group$^{\Delta}$, 2, 8, and 12% for 0, 8,000, and 20,000 ppm M groups).
**S-D rats (n =50 M and 50 F)**	Diet, 0, 30, 100, or 300 ppm	Life-time, up to 26 months	Pancreas islet cell adenoma (10% in the 30 ppm M group$^{\Delta}$, 0, 4, and 4% for 0, 100, and 300 ppm M groups).
**Wistar rats (n =50 M and 50 F)**	Water, 0, 300, 900, or 2,700 mg/L	24 months	NS
**S-D rats (n =50 M and 50 F)**	Diet, 0, 10, 100, 300, or 1,000 mg/kg bw	24 months	NS
**Wistar-Alpk:ApfSD (n =52 M and 52 F)**	Diet, 0, 2,000, 6,000, or 20,000 ppm	24 months	NS
**Wistar-Alpk:ApfSD (n =24 M and 24 F)**	Diet, 0, 2,000, 8,000, or 20,000 ppm	12 months	NS

* M, male; F, female. S-D, Sprague-Dawley

** All treatments were *ad libitum*

*** Only tumors with a statistically significant result are shown with the dosage and % underlined ($^{\Delta}$, $P \leq 0.05$ compared to the no glyphosate group; bold and underlined denotes no furthur increase in incidence at highest doses). NS, not significant increase.
